# Glucosylceramidase Maintains Influenza Virus Infection by Regulating Endocytosis

**DOI:** 10.1128/JVI.00017-19

**Published:** 2019-05-29

**Authors:** Kelly Drews, Michael P. Calgi, William Casey Harrison, Camille M. Drews, Pedro Costa-Pinheiro, Jeremy Joseph Porter Shaw, Kendra A. Jobe, Elizabeth A. Nelson, John D. Han, Todd Fox, Judith M. White, Mark Kester

**Affiliations:** a Department of Pathology, University of Virginia, Charlottesville, Virginia, USA; b Department of Biomedical Engineering, University of Virginia, Charlottesville, Virginia, USA; c Department of Environmental Sciences, University of Virginia, Charlottesville, Virginia, USA; d Department of Cell Biology, University of Virginia, Charlottesville, Virginia, USA; e Department of Biology, University of Virginia, Charlottesville, Virginia, USA; f Department of Pharmacology, University of Virginia, Charlottesville, Virginia, USA; g Department of Microbiology, University of Virginia, Charlottesville, Virginia, USA; University of Kentucky College of Medicine

**Keywords:** endocytosis, glycolipids, influenza, vesicular trafficking, virology, virus entry

## Abstract

Influenza virus is an RNA virus encapsulated in a lipid bilayer derived from the host cell plasma membrane. Previous studies showed that influenza virus infection depends on cellular lipids, including the sphingolipids sphingomyelin and sphingosine. Here we examined the role of a third sphingolipid, glucosylceramide, in influenza virus infection following clustered regularly interspaced short palindromic repeats with Cas9 (CRISPR-Cas9)-mediated knockout (KO) of its metabolizing enzyme glucosylceramidase (GBA). After confirming GBA knockout of HEK 293 and A549 cells by both Western blotting and lipid mass spectrometry, we observed diminished infection in both KO cell lines by a PR8 (H1N1) green fluorescent protein (GFP) reporter virus. We further showed that the reduction in infection correlated with impaired influenza virus trafficking to late endosomes and hence with fusion and entry. To examine whether GBA is required for other enveloped viruses, we compared the results seen with entry mediated by the glycoproteins of Ebola virus, influenza virus, vesicular stomatitis virus (VSV), and measles virus in GBA knockout cells. Entry inhibition was relatively robust for Ebola virus and influenza virus, modest for VSV, and mild for measles virus, suggesting a greater role for viruses that enter cells by fusing with late endosomes. As the virus studies suggested a general role for GBA along the endocytic pathway, we tested that hypothesis and found that trafficking of epidermal growth factor (EGF) to late endosomes and degradation of its receptor were impaired in GBA knockout cells. Collectively, our findings suggest that GBA is critically important for endocytic trafficking of viruses as well as of cellular cargos, including growth factor receptors. Modulation of glucosylceramide levels may therefore represent a novel accompaniment to strategies to antagonize “late-penetrating” viruses, including influenza virus.

**IMPORTANCE** Influenza virus is the pathogen responsible for the second largest pandemic in human history. A better understanding of how influenza virus enters host cells may lead to the development of more-efficacious therapies against emerging strains of the virus. Here we show that the glycosphingolipid metabolizing enzyme glucosylceramidase is required for optimal influenza virus trafficking to late endosomes and for consequent fusion, entry, and infection. We also provide evidence that promotion of influenza virus entry by glucosylceramidase extends to other endosome-entering viruses and is due to a general requirement for this enzyme, and hence for optimal levels of glucosylceramide, for efficient trafficking of endogenous cargos, such as the epidermal growth factor (EGF) receptor, along the endocytic pathway. This work therefore has implications for the basic process of endocytosis as well as for pathogenic processes, including virus entry and Gaucher disease.

## Abstract (precis)

Influenza virus is the pathogen responsible for the second largest pandemic in human history. A better understanding of how influenza virus enters host cells may lead to the development of more-efficacious therapies against emerging strains of the virus. Here we show that the glycosphingolipid metabolizing enzyme glucosylceramidase is required for optimal influenza virus trafficking to late endosomes and for consequent fusion, entry, and infection. We also provide evidence that promotion of influenza virus entry by glucosylceramidase extends to other endosome-entering viruses and is due to a general requirement for this enzyme, and hence for optimal levels of glucosylceramide, for efficient trafficking of endogenous cargos, such as the epidermal growth factor (EGF) receptor, along the endocytic pathway. This work therefore has implications for the basic process of endocytosis as well as for pathogenic processes, including virus entry and Gaucher disease.

## INTRODUCTION

Between three and five million people are infected with influenza A virus (IAV) worldwide each year, with one quarter million to half a million cases resulting in death. While therapies against influenza exist, they are often administered too late to provide patient relief. Vaccines against the virus are produced each year but may provide limited coverage against isolates arising from antigenic shift, such as occurred during the 2009 H1N1 pandemic, which is estimated to have killed up to 575,000 people (1). IAV is a negative-sense RNA virus belonging to the family *Orthomyxoviridae* and is an enveloped virus that derives its lipid bilayer membrane as the virus buds through the host plasma membrane during virus assembly. To infect a cell, influenza virus employs its hemagglutinin (HA) protein to bind to sialic acid moieties on the target cell surface and is then taken into the cell by endocytosis (2). As the virus travels along the endocytic pathway, the acid environment prevailing in endosomes prompts conformational changes in HA, leading to viral membrane fusion with a late endosomal membrane (at pH ∼5.0 to 5.7, depending on the strain) and subsequent genome release into the cytoplasm to initiate replication (3–7). Hence, proper endosomal trafficking and pH are crucial to the influenza virus life cycle (8–10).

**FIG 1:**
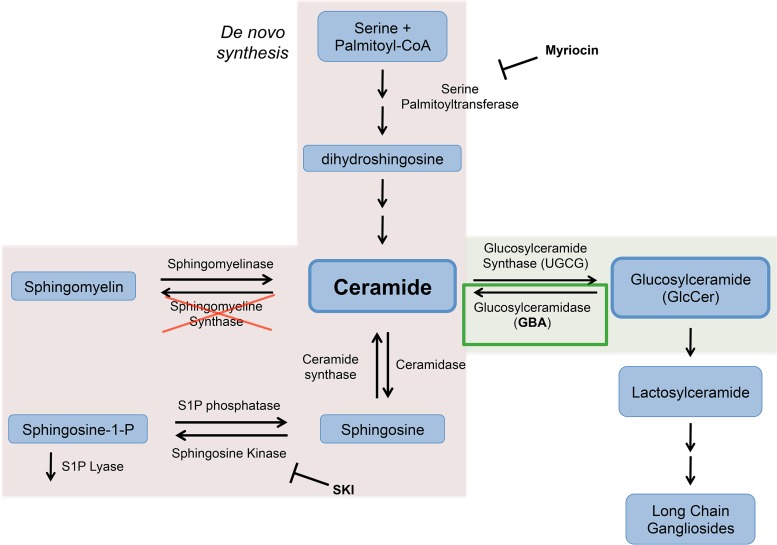
Role of sphingolipids in influenza virus infection. The sphingolipid pathway involves numerous enzymes and lipids, most of which shuttle through ceramide as the pathway hub. Previous studies showed that deficiencies in sphingomyelin synthase and inhibition of serine palmitoyltransferase or sphingosine kinase led to decreased levels of influenza virus infection (red shading) (23–25). However, the glycosphingolipid arm of the sphingolipid pathway (green shading) has not yet been studied in the context of influenza virus. We specifically examined glucosylceramidase (green box) to determine the effects of glucosylceramide metabolism on influenza virus infections.

The membrane of influenza virus contains sphingolipids, which are members of a class of bioactive signaling molecules broadly distributed in mammalian cells and integral to multiple cell functions (11). Sphingolipids have also been shown to play diverse roles in virus-host interactions (12), including promoting virus binding (13–16), entry (17–19), replication (20, 21), and new particle release (22). Several laboratories have explored the relationship between influenza virus and sphingolipids, notably sphingosine-1-phosphate (S1P) and sphingomyelin (Fig. 1). Overexpression of S1P lyase reduced influenza virus infection whereas overexpression of sphingosine kinase increased infection in host cells (23). Moreover, influenza virus infection was shown to activate sphingosine kinase, generating sphingosine-1-phosphate, which was shown to increase viral RNA synthesis and nuclear export of influenza virus ribonucleoprotein complexes (24). Cells deficient in sphingomyelin synthase displayed reduced transport of the influenza virus glycoproteins (HA and neuraminidase [NA]) to the cell surface, and pharmacological reduction of sphingomyelin with myriocin led to decreased influenza virus infection (25). Those studies suggested that sphingolipid metabolism may provide an important target for discovery of future influenza therapeutics.

At the hub of sphingolipid metabolism is ceramide, an apoptosis-inducing molecule that can be modified at both its polar head group and its carbohydrate chain to generate numerous sphingolipid species (26) (Fig. 1). Ceramide is converted to the glycosphingolipid glucosylceramide (GlcCer) by the addition of a glucose moiety catalyzed by the enzyme glucosylceramide synthase (UGCG), which is found primarily in the Golgi compartment (27). In contrast, catabolism of GlcCer to remove the glucose group is performed by glucosylceramidase (GBA), which is reported to be primarily found in lysosomes (27). Mutations in GBA are well-studied genetic determinates of Gaucher disease, one of the most common lysosomal storage disorders (28, 29). While GBA has been implicated in trafficking of membrane glycolipids along the endocytic pathway (30), there have not been studies on the role of GBA in endocytic cargo trafficking. Moreover, while several studies have focused on the conversion of sphingomyelin to ceramide in the context of viral infections (Fig. 1; pink shading) (13, 17, 18, 30–32), little attention has been focused on the glucosylceramide arm of sphingolipid metabolism (Fig. 1; green shading). One recent study employing a haploid genetic screen revealed a role for UGCG in infections by specific bunyaviruses but not in those by other viruses tested (19). And, to our knowledge, no one has investigated the role of GBA in any viral infection.

In this study, we explored the role of GBA in influenza virus entry and infection by genetically knocking it out using clustered regularly interspaced short palindromic repeats with Cas9 (CRISPR-Cas9). We found that cells deleted for GBA displayed reduced influenza virus trafficking to late endosomes and consequent fusion, entry, and infection, suggesting that GBA and, by extension, optimal levels of its substrate lipid, GlcCer, are critical for maintaining the influenza life cycle in host cells. We also provide evidence that GBA is required for the entry of other viruses that enter cells by endocytosis as well as for the proper trafficking and disposition of normal cellular vesicular cargos destined for late endosomes, including epidermal growth factor (EGF) and its receptor (EGFR).

## RESULTS

### Glucosylceramide metabolism regulates influenza virus infection.

Previous studies have demonstrated that certain enzymes along the sphingolipid pathway **(**Fig. 1) are important for influenza virus infection; cells exposed to inhibitors of serine palmitoyltransferase or sphingosine kinase, as well as cells deficient in sphingomyelin synthase, displayed reduced influenza virus infection (23–25). The role of glucosylceramidase (GBA) or its substrate, glucosylceramide (GlcCer), has not been explored in influenza virus infections. To examine the role of GBA in influenza virus infection, we generated knockout (KO) cell lines lacking GBA using clustered regularly interspaced short palindromic repeats with Cas9 (CRISPR/Cas9) gene editing. CRISPR/Cas9 was performed in two cell lines, human embryonic kidney (HEK) 293 and adenocarcinomic lung epithelial A549. HEK 293 cells were chosen for their ease of use and their broad use in cell biology, while A549 cells were chosen as they, being derived from lung epithelia, are considered more physiologically relevant for influenza research.

**FIG 2:**
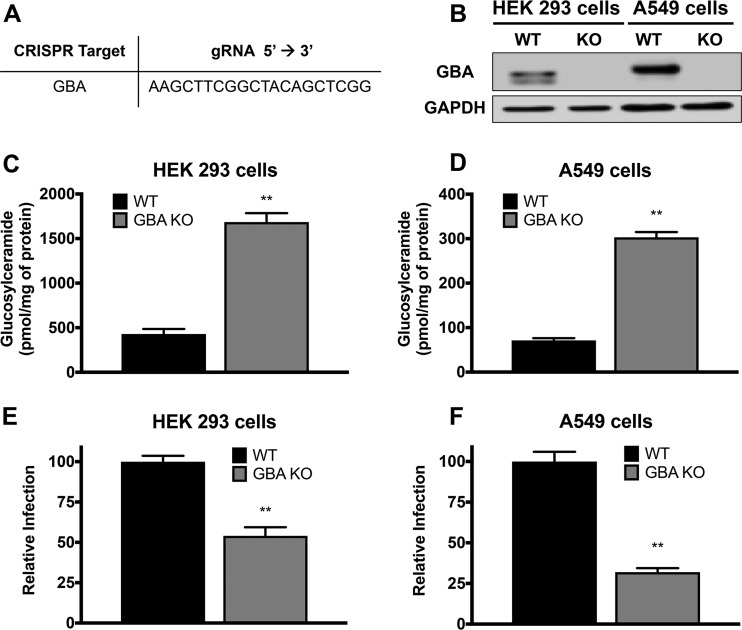
Glucosylceramidase regulates influenza virus infection. HEK 293 and A549 cells were transfected with plasmids encoding Cas9-sgRNA targeting GBA and a plasmid containing GFP. Single cell colonies were selected for successful transfection as measured by GFP expression and expanded. (A) The gRNA used to target GBA is listed. (B) Complete loss of GBA protein expression was confirmed by Western blot analysis of lysates of both HEK 293 and A549 cell colonies. (C and D) Lipids were extracted from the cells and analyzed by mass spectrometry. Consistent with KO status, total GlcCer levels were raised in both HEK 293 (C) and A549 (D) GBA knockouts. Data represent the mean values of six biological replicates ± standard errors (SE). Loss of GBA activity was confirmed using a direct enzyme assay (data not shown). (E and F) Cells in triplicate samples were infected at 4°C with PR8 influenza virus encoding an NS1-GFP chimeric protein and were then incubated for ∼18 h at 37°C. The cells were then harvested, fixed, and analyzed for GFP expression by flow cytometry. In both the HEK 293 (E) and A549 (F) cell lines, GBA knockout resulted in decreased influenza virus infection compared to WT as measured by NS1-GFP expression. Data represent means ± SE, *n* = 6 experiments. **, *P* < 0.01 (using a Mann-Whitney nonparametric test). The growth rates of WT and GBA KO cells were similar over the duration studied (up to 4 days) (data not shown).

After CRISPR/Cas9 gene editing (Fig. 2A) and isolation of individual clones were performed, cells were analyzed for expression of GBA by Western blot analysis and for the resulting concentration of GlcCer by mass spectrometry. In both HEK 293 and A549 cells, CRISPR/Cas9 targeting resulted in complete loss of GBA protein as detected by Western blotting (Fig. 2B). Mass spectrometry revealed that in both HEK 293 and A549 cells, GBA KO resulted in approximately 3-fold to 4-fold increases in GlcCer levels (Fig. 2C and D).

After confirming functional KO of GBA in the two cell lines described above, we examined the effect of loss of GBA on influenza virus infection. As seen in Fig. 2E and F, GBA KO in HEK 293 cells resulted in an ∼50% decrease in PR8 influenza virus infection, while GBA KO in A549 cells resulted in an ∼70% decrease.

### Changes in sphingolipid species in GBA KO cells.

**FIG 3:**
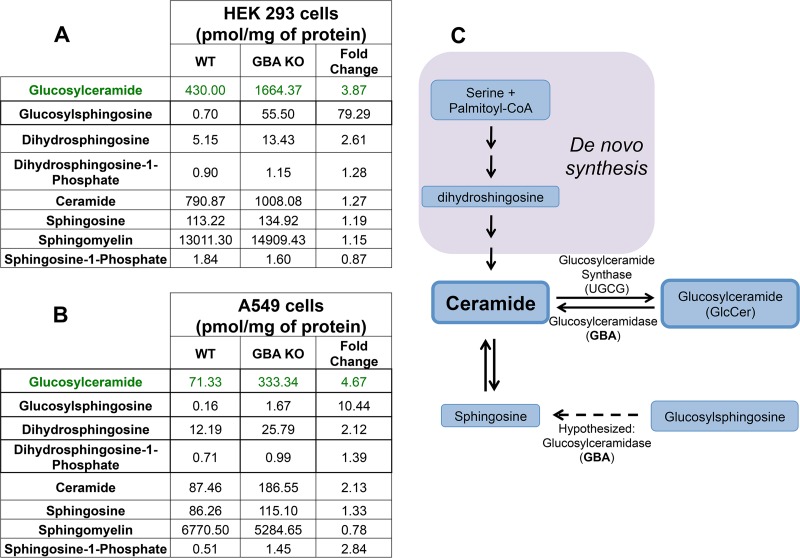
Analysis of lipid levels in GBA knockout cells. (A and B) Sphingomyelin, ceramide, glucosylceramide, glucosylsphingosine, sphingosine, and sphingosine-1-phosphate were analyzed by mass spectrometry in all knockout cells and compared to their WT counterparts. Mass spectrometry peaks were compared to internal standards, and all data are represented as picomoles of lipid/milligram of protein (mean values shown; *n* = 6). The data for GlcCer are the same as those displayed graphically in Fig. 2C and D. (C) Mass spectrometry data indicate that GBA may utilize glucosylsphingosine as a secondary substrate (indicated by dashed arrow). *De novo* synthesis of ceramide is indicated by purple shading.

As noted above, loss of GBA resulted in an expected increase in GlcCer levels. Surprisingly, this was not accompanied by a corresponding decrease in ceramide levels (Fig. 3A and B). Moreover, major changes in the levels of downstream ceramide metabolites such as sphingosine-1-phosphate and sphingomyelin were not noted. Taking the results together, we hypothesize that the lack of change in ceramide levels was due to compensatory *de novo* synthesis of ceramide, consistent with the increase seen in the levels of dihydrosphingosine (Fig. 3A and B), an intermediate metabolite in *de novo* synthesis (Fig. 1 and Fig. 3C; in purple). Interestingly, GBA KO cells displayed an even greater fold change in glucosylsphingosine levels (Fig. 3A and B), although the total mass of glucosylsphingosine is hundred-folds lower than that of glucosylceramide. Little is known regarding the metabolism of glucosylsphingosine, but the increase in its level suggests a potential new role for GBA in the catabolism of glucosylsphingosine (Fig. 3C).

### Glucosylceramidase regulates entry of influenza virus and other endocytosed viruses.

**FIG 4:**
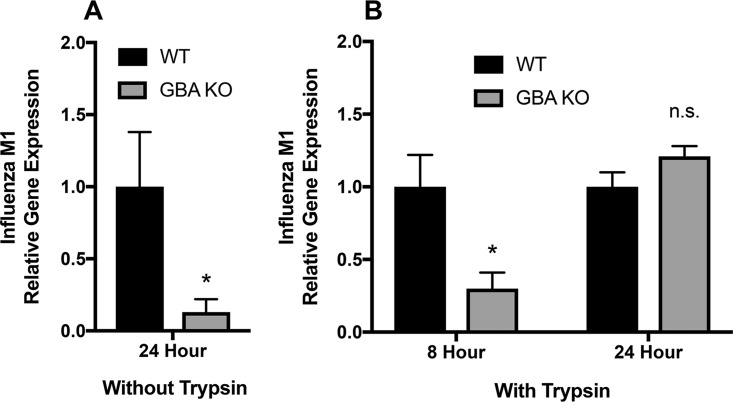
Influenza virus M1 gene expression is time and trypsin dependent in GBA KO cells. WT and GBA KO cells in triplicate samples were incubated at 4°C with PR8 influenza virus and then incubated for 8 or 24 h at 37°C with or without trypsin in the medium. Samples were collected, and mRNA was extracted, cDNA was generated, and relative gene expression levels were analyzed by qPCR. (A) GBA KO cells displayed a reduction in influenza virus M1 expression when incubated without trypsin and were therefore limited to one cycle of influenza virus infection. (B) Influenza virus M1 expression was reduced after 8 h in GBA KO cells in the presence of trypsin, the level of expression matched that of WT cells but after 24 h. n.s., not statistically significant.

The reduction in influenza virus infection observed in GBA KO cells (Fig. 2E and F) could be due to defects at different stages of the viral life cycle. To begin to identify the step requiring GBA, we first analyzed IAV matrix protein 1 (M1) mRNA after 24 h in cells incubated in media lacking trypsin, in order to limit IAV infections to one cycle of replication. GBA KO cells exhibited reduced IAV M1 expression after 24 h (Fig. 4A). We next analyzed IAV M1 mRNA at two time points postinfection in the presence of trypsin (to cleave the HA precursor and therefore permit production of infectious particles). After 8 h, GBA KO cells displayed reduced IAV M1 expression compared to wild-type (WT) cells, but no difference was seen at 24 h (Fig. 4B). These data suggest that the observed reduction in influenza virus infection represented in Fig. 2E and F was limited to one cycle of replication, likely at the level of virus entry.

**FIG 5:**
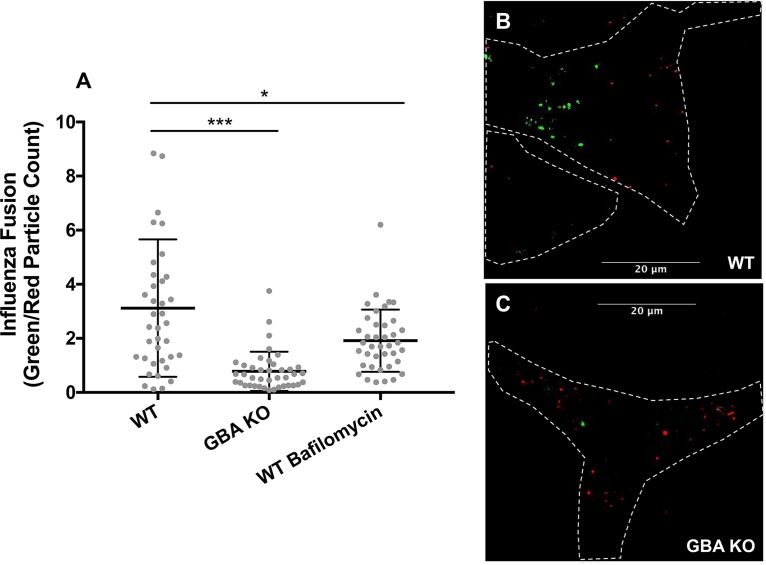
Loss of GBA reduces influenza virus fusion in endosomes. Influenza virus was labeled with R18 (red) and DiOC18 (green) and then added to prechilled A549 cells at 4°C for 15 min. Cells were then washed, incubated at 37°C for 30 min, fixed, and imaged at ×60 magnification. The numbers of green particles (indicating a fused virus) and red particles (indicating an unfused virus) were then analyzed using ImageJ particle analysis. WT cells pretreated with 100 nM bafilomycin, an endosome acidification inhibitor, for 1 h served as a positive control. (A) The ratio of fused particles to unfused particles was measured by automated counting of the number of green particles divided by the number of red particles on an image-by-image basis using images from 2 experiments (*n* = 40 fields for each treatment). Each data point represents the total number of green particles divided by the total number of red particles for 1 image field, and the bars indicate mean values ± standard deviations (SD). (See Materials and Methods for details.) (B and C) Representative WT (B) and GBA KO (C) images. Cell outlines were marked by visual examination. *, *P* < 0.05; ***, *P* < 0.001 (using a Mann-Whitney nonparametric test).

To test whether GBA regulates influenza virus entry (through endosomes), we monitored fusion of A/PR/8/34 influenza (H1N1) virus labeled with octadecyl rhodamine B chloride (R18) and 3,3′-dioctadecyloxacarbocyanine (DiOC18). Fusion resulted in a shift in fluorescence emission (EM) from red (R18, wavelength of 586 nm) to green (DiOC18, 510 nm) due to separation of the probes upon fusion and dilution into the endosome membrane (33, 34). As shown in Fig. 5A, there were ∼3.5 fused influenza virus particles for every nonfused particle in the WT cells, while the corresponding ratio in the GBA KO cells was less than 1. The results presented in Fig. 5 indicate that GBA is necessary for influenza virus particles to fuse in endosomes and suggest that the observed reduction in influenza virus infection (Fig. 2E and F and Fig. 4) was due to a defect in the entry phase of the viral life cycle.

**FIG 6:**
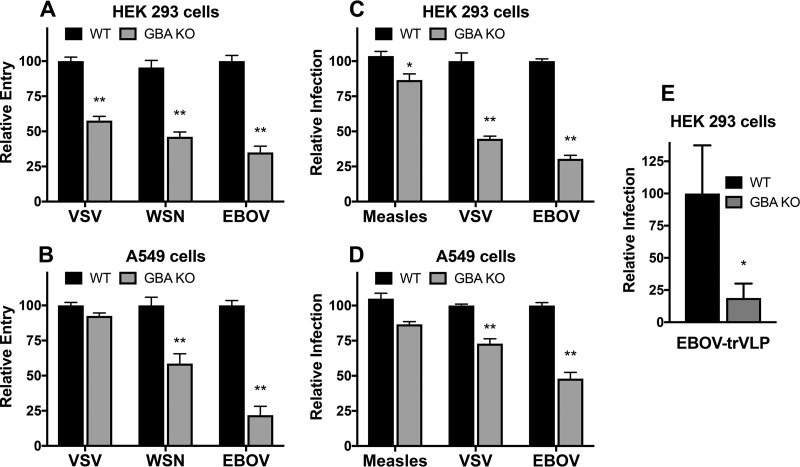
Loss of GBA reduces entry mediated by the glycoproteins of other endosome-entering enveloped viruses, with minimal effects on entry mediated by the glycoproteins of measles virus, a plasma-membrane-entering virus. (A and B) Influenza virus-like particles (VLPs) bearing the VSV-G, WSN HA/NA, or EBOV-GPΔ glycoproteins were generated on a βlaM1 backbone as described in Materials and Methods. VLPs were added to prechilled cells, and the complexes were centrifuged at 4°C for 1 h, incubated for 3 h at 37°C, and then incubated for 1 h at room temperature in the dark in the presence of a fluorescent β-lactamase substrate. Cells were washed and the following day were harvested, fixed, and analyzed for β-lactamase activity (corresponding to relative entry [A] and relative infection [B]) via flow cytometry. (See Materials and Methods for details.) Data represent means ± SE; *n* = 6 experiments. (C and D) VSV pseudoviruses bearing the measles virus F and HN, VSV-G, or EBOV-GPΔ glycoproteins and encoding GFP were generated and bound to prechilled cells by centrifugation at 4°C for 1 h. The pseudovirus-cell complexes were then incubated at 37°C for 24 h, after which they were lifted, fixed, and analyzed for GFP expression (corresponding to relative entry [C] and relative infection [C]) via flow cytometry. Data represent means ± SE; *n* = 6 experiments. *, *P* < 0.05; **, *P* < 0.01 (using a Mann-Whitney nonparametric test). (E) WT and GBA KO HEK 293 cells were infected with EBOV trVLPs for 24 h at 37°C, and infection was assayed as described in Materials and Methods. Data represent means ± SE; *n* = 5. *, *P* < 0.05 (using a Mann-Whitney nonparametric test).

We next investigated whether entry of other endocytosed enveloped viruses is impacted by loss of GBA. To do this, we generated virus-like particles (VLPs) with an influenza virus matrix protein 1 (M1)–β-lactamase (β-lam) (βlaM1) core and bearing different viral glycoproteins on their surfaces using the following: vesicular stomatitis virus G protein (VSV-G), which directs fusion in early endosomes (pH ∼6.0); WSN influenza virus HA, which directs fusion in late endosomes (pH ∼5.0 to 5.5); and Ebola virus glycoprotein (EBOV-GP), which directs fusion in endolysosomes (pH ∼4.5 to 5.0) (34–36). WT and GBA KO cells were incubated with the VLPs and assayed for VLP entry using a fluorescent β-lam substrate in conjunction with flow cytometry (Fig. 6A and B). The level of entry mediated by VSV-G was reduced in HEK 293 GBA KO cells but was unaffected in the corresponding A549 KO cells. Entry mediated by the HA of WSN influenza virus was reduced in both the HEK 293 and A549 GBA KO cells, consistent with the PR8 infection (Fig. 2E and F and Fig. 4) and fusion (Fig. 5) data. The level of entry mediated by EBOV-GP was also reduced in both KO cell lines and the extent of entry trended toward a lower level than entry mediated by the glycoproteins of WSN influenza virus or VSV. These findings suggest that other viruses that enter cells through endosomes depend on GBA and further support the hypothesis of a greater entry inhibition for viruses that fuse with later endosomes, suggesting that GBA may affect endosome maturation and/or acidification. The differing results for VSV-G-VLPs between A549 and HEK 293 GBA KO cells may indicate cell type dependence for GBA in the early endocytic pathway.

To further explore the breadth of viruses whose entry is affected by GBA, we employed pseudoviruses with a VSV core and displaying the glycoproteins of measles virus, which fuses at neutral pH at the cell surface, or displaying the glycoproteins of the VSV and EBOV, which enter cells through endosomes. The levels of infections by VSV pseudoviruses bearing the VSV and EBOV glycoproteins were decreased in GBA KO cells (Fig. 6C and D), as seen with their corresponding influenza virus M1-VLPs (Fig. 6A and B). In contrast, infection by pseudoviruses bearing the glycoproteins of measles virus appeared less dependent on GBA. These findings support the contention that virus entry that occurs through the activity of endosomes, particularly through that of late endosomes, is dependent on the presence of functional GBA and, by extension, of optimal levels of GlcCer. Interestingly, we tested the role of GBA in EBOV infections using EBOV transcription/replication-competent VLPs (trVLPs), which recapitulate the full Ebola virus life cycle and can be used under biosafety level 2 (BSL2) conditions (37). As seen in Fig. 6E, loss of GBA strongly reduced Ebola trVLP infection even after multiple cycles of replication. These trVLP data contrast with the finding represented in Fig. 4 that indicates that the effect of GBA on influenza virus infection is limited to viral entry. Taking the results together, the Fig. 4 and Fig. 6E data suggest that the role of GlcCer in viral assembly and/or budding may be virus specific.

**FIG 7:**
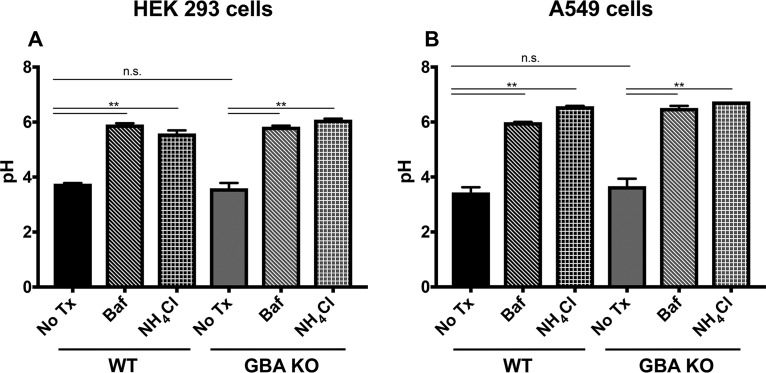
Loss of GBA does not detectably alter lysosomal pH. Cells were incubated with FITC-dextran for 72 h followed by a 2-h pulse in medium without FITC-dextran. Cells were pretreated with bafilomycin (Baf) or NH_4_Cl where indicated for 1 h at 37°C to serve as positive controls. No Tx, no treatment. Following the dextran-free pulse, cells were analyzed by flow cytometry. Samples were compared to a standard curve of pH controls to determine lysosomal pH, as described in Materials and Methods. Data represent means ± SE; *n* = 5 experiments.

As the observed decreases in entry by viruses that fuse in late endosomes could have been due to a defect in endosome acidification, we measured the pH of endosomes in GBA KO cells. To accomplish this, we employed a dual-emission ratiometric technique after feeding cells fluorescein isothiocyanate (FITC)-dextran (38). We utilized two independent positive controls (bafilomycin and NH_4_Cl) to confirm changes in pH. Using this technique, we determined that the pH in endosomes in GBA KO cells was not detectably different than that in WT cells (Fig. 7). These data suggest that our observed viral entry phenotype is not due to changes in the acidification process of the endosome.

### Glucosylceramidase regulates influenza virus trafficking along the endocytic pathway.

**FIG 8:**
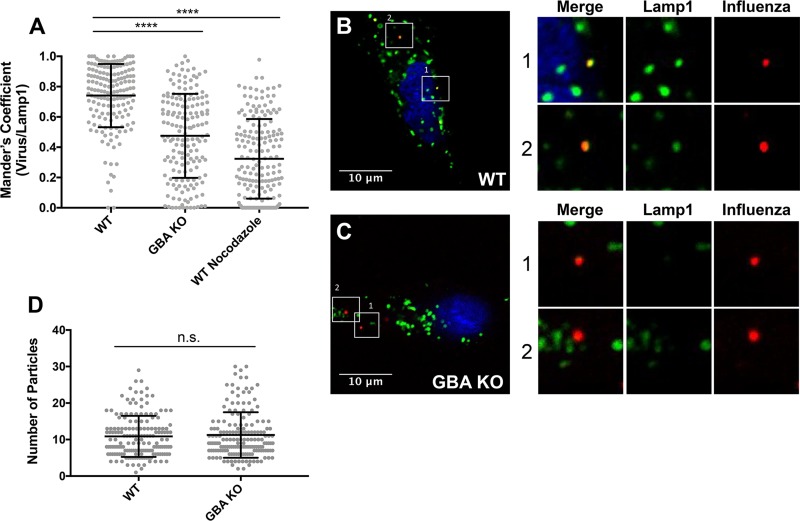
Trafficking of influenza virus to late endosomes is impaired in GBA KO cells. A549 cells were transfected with Lamp1-GFP 1 day prior to experiments. WT cells were pretreated with 40 μM nocodazole (as a positive control) for 1 h where indicated. Influenza virus (PR8) was labeled with R18 and then added to prechilled cells at an MOI of ∼10 at 4°C for 15 min. Cells were washed, incubated at 37°C for 40 min, fixed, and imaged at ×100 magnification. (A) Average Manders colocalization coefficients of influenza with Lamp1 (± SD) from 2 experiments (*n* = 100 fields in each experiment). Each data point represents the Manders colocalization coefficient for 1 image field. (B and C) Representative micrographs of cells infected with R18-labeled influenza virus. Numbered white boxes are shown as enlarged images to the right of each panel. Examples are of colocalized particles in the WT cells (B) and of noncolocalized particles in the knockout cells (C). (D) Total number of influenza virions in each image analyzed as described for panel A. ****, *P* < 0.0001 (based on a Mann-Whitney nonparametric test).

It is well established that influenza virus traffics to late endosomes for fusion (39). In addition, GlcCer has been implicated in lipid transport along the endocytic pathway (40). We therefore asked if GBA is important for trafficking of influenza virus particles to late endosomes. We transfected WT and GBA KO A549 cells with Lamp1-GFP (green fluorescent protein) and then infected the cells with R18-labeled influenza virus (red). In WT cells at 40 min postwarming, influenza virus could be visualized in Lamp1^+^ (green) endosomes in a high percentage of cells (Fig. 8A and B). In contrast, colocalization was reduced in GBA KO cells (Fig. 8A and C), albeit not as strongly as in WT cells treated with nocodazole (Fig. 8A), a microtubule inhibitor known to block trafficking between early and late endosomes. The observation that equivalent numbers of influenza virus particles were seen in WT and GBA KO cells (Fig. 8D) indicates that there is not a defect in influenza binding to GBA KO cells but that there is instead a defect in trafficking along the endocytic pathway.

### Glucosylceramidase regulates epidermal growth factor receptor trafficking along the endocytic pathway.

**FIG 9:**
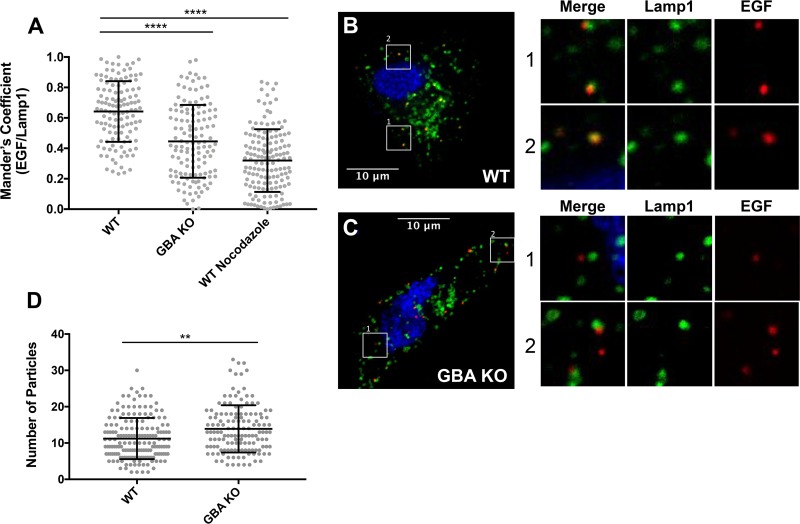
Trafficking of EGF to late endosomes is impaired in GBA KO cells. A549 cells were transfected with Lamp1-GFP and pretreated with nocodazole as described for Fig. 6. EGF-555 (100 ng/ml) was added to cells prechilled to 4°C for 15 min. Cells were then washed, incubated at 37°C for 40 min, fixed, and imaged. (A) Average Manders colocalization coefficients of EGF with Lamp1 (± SD) from 2 experiments (*n* = 100 fields in each experiment). Each data point represents the Manders colocalization coefficient for 1 image field. (B and C) Representative micrographs of cells incubated with EGF-555. White boxes are shown as enlarged images to allow better qualitative visualization. Examples of colocalized particles pictured in the WT cells (B) and of noncolocalized particles pictured in the knockout cells (C) are shown. (D) Total number of EGF particles in each image analyzed as described for panel A. **, *P* < 0.01; ****, *P* < 0.0001 (using a Mann-Whitney nonparametric test).

In view of the findings on influenza virus trafficking, we next asked if GBA is required for proper trafficking of nonviral cargo along the endocytic pathway. We utilized the same approach as that depicted in Fig. 8 but used fluorescently tagged epidermal growth factor (EGF) (red) (Fig. 9). Similarly to the influenza virus results, at 40 min postwarming, EGF was seen in a high percentage of Lamp1^+^ (green) endosomes in WT cells, while colocalization was reduced in GBA KO cells. As with the influenza virus, the decrease in colocalization of EGF with Lamp1 was not as severe as that seen in WT cells treated with nocodazole (Fig. 9A). There was no decrease in the number of EGF particles in GBA KO versus WT cells (Fig. 9D; a small increase was seen), indicating a postbinding requirement for GBA for proper trafficking of EGF.

**FIG 10:**
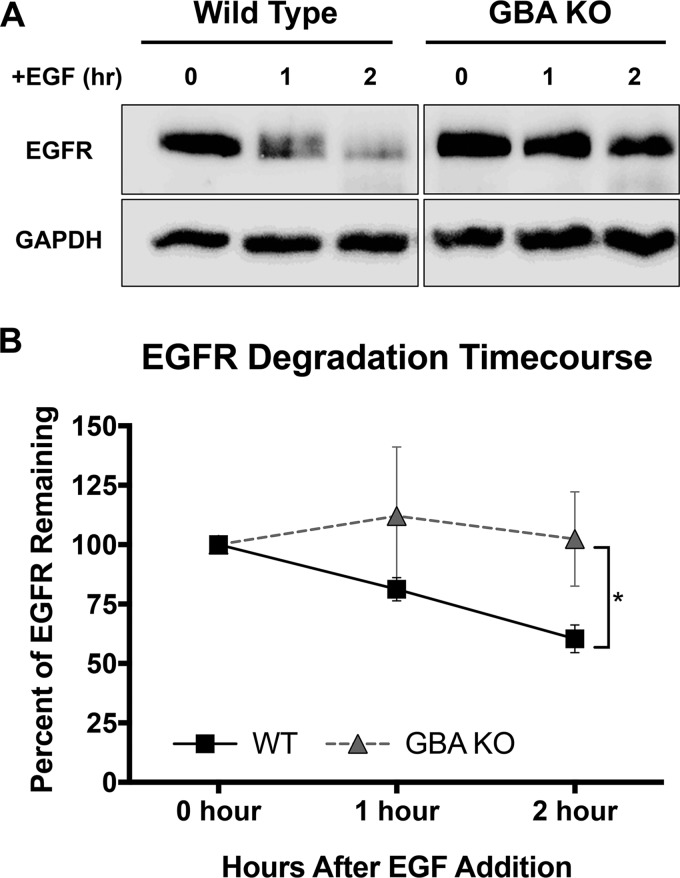
EGFR degradation is impaired in GBA KO Cells. EGF (50 ng/ml) was added to cells, and the cells were incubated at 37°C. At the indicated times, cell lysates were prepared and subjected to SDS-PAGE and Western blotting for EGFR. (A) Representative Western blots. (B) Quantitation of Western blots indicated significant differences in the levels of EGFR remaining at 2 h postaddition of EGF in GBA KO cells compared to WT cells. Values are normalized to the intensity of GAPDH and are presented as percentages of EGFR remaining compared to cells without EGF stimulation. Data represent means ± SE; *n* = 6 experiments. *, *P* < 0.05 (using a Mann-Whitney nonparametric test).

Following binding of EGF to its receptor (EGFR) at the cell surface, the EGF-EGFR complex is transported to lysosomes and degraded by proteases, including cathepsins (Cats) (41). Since EGF trafficking was impaired in GBA KO cells, we investigated whether degradation of EGFR was been impaired. To do this, medium containing EGF was added to cells at 37°C, and at various times the cells were harvested, lysed, and analyzed for the presence of EGFR by Western blot analysis. As shown and quantified in Fig. 10, and consistent with the EGF trafficking data (Fig. 9), degradation of EGFR was notably impaired in GBA KO cells.

**FIG 11:**
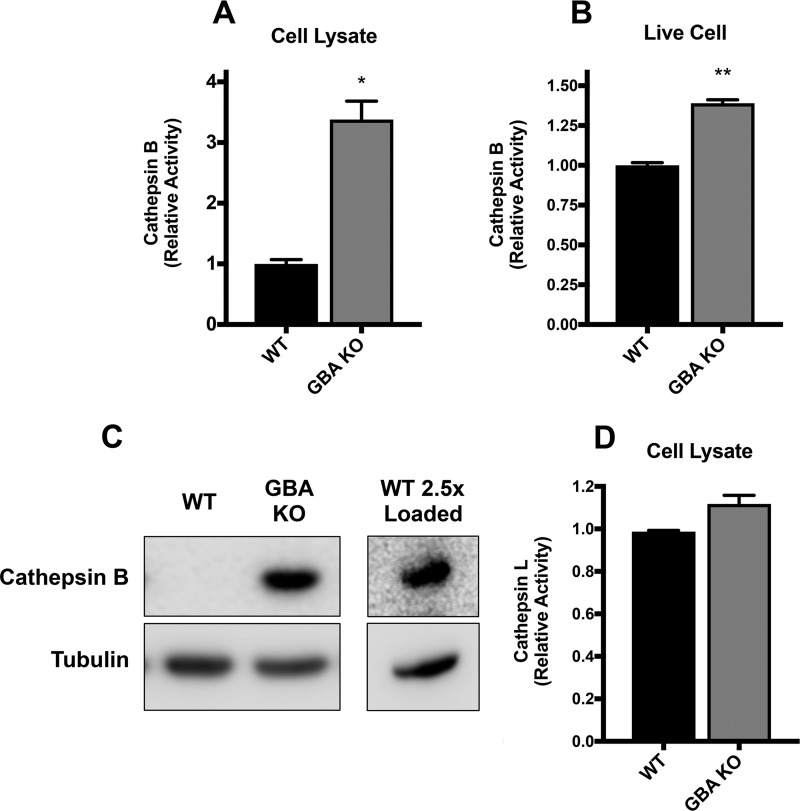
Loss of GBA upregulates cathepsin B activity. (A) WT and GBA KO A549 cells were lysed and analyzed for cathepsin B activity following incubation with a cathepsin B-specific substrate for 1 h at 37°C, as described in Materials and Methods. Data represent means ± SE; *n* = 5 experiments. (B) *In vivo* cathepsin B activity in A549 cells was determined using the Magic Red-(RR)_2_ cathepsin activity assay per the instructions of the manufacturer. Data represent means ± SE; *n* = 5 experiments. (C) Cathepsin B was probed by Western blotting and found to be undetectable in A549 WT samples but readily detectable in GBA KO cells. Loading 2.5× the amount of lysate resulted in CatB detection in the WT cells. (D) WT and GBA KO cells were lysed and processed as described for panel A were but incubated with a cathepsin L-specific substrate and analyzed for cathepsin L activity as described in Materials and Methods. *, *P* < 0.05; **, *P* < 0.01 (using a Mann-Whitney nonparametric test).

We proposed that the defect in EGFR degradation in GBA KO cells (Fig. 10) was due to a defect in EGFR trafficking (Fig. 9) as opposed to a defect in endosome acidification (Fig. 7) or in cathepsin activities. To test the latter possibility, we examined the levels of two cysteine proteases normally found in lysosomes, cathepsin B (CatB) and CatL. This was done *in vitro*, following cell lysis and adjustment to an acidic pH for CatB and CatL by the use of fluorescent peptide substrates, and, additionally, for Cat B, in live cells using Magic Red-(RR)_2_ (42). Unexpectedly, the GBA KO cells displayed an ∼3.5-fold increase in CatB activity compared to the WT cells in the *in vitro* assay (Fig. 11A). CatB activity was also increased in the live cell assay, but the increase was not as great (Fig. 11B). Consistently, we observed an increase in CatB protein levels in GBA KO cells (Fig. 11C). CatL activity in cell lysates displayed no significant differences between the KO cells and the WT cells (Fig. 11D). Taken together, these data suggest that diminished cathepsin expression and/or activity was not the cause of dysfunctional EGFR degradation in GBA KO cells and further support our proposed mechanism of impaired endosome trafficking. The mechanism behind the unexpected increase in CatB activity and expression in GBA KO cells may have been the result of defects in CatB localization and warrants further investigation.

In summary, our findings suggest that GBA and optimal levels of GlcCer are required to regulate trafficking along the endocytic pathway of viruses and endogenous cargos, particularly with respect to later stages of the pathway. Consequently, when GBA is missing, endocytosed enveloped viruses show diminished fusion, entry, and infection and critical growth factor receptors are not degraded, a process required for proper growth control. Collectively, the findings presented in Fig. 7 and 11 indicate that the defect in viral entry (and consequent infection) and EGFR degradation seen in the GBA KO cells was not due to lowered levels of cathepsins or impaired endosome acidification but rather to a defect in trafficking of cargos to degradative endosomes/lysosomes.

## DISCUSSION

In this study, we established a role for glucosylceramidase (GBA) in the regulation of endocytosis of viral and cellular cargos, first for endosomal entry and infection by influenza virus. We established that the defect in influenza virus infection in GBA KO cells is due to defects in delivery to late endosomes and consequent fusion and entry into the cytoplasm. Consistently, we observed defects in entry mediated by the glycoproteins of other enveloped viruses that enter cells through endosomes (4); VLPs and pseudoviruses bearing VSV, influenza virus, and Ebola (EBOV) glycoproteins displayed decreased entry into GBA KO cells, with minimal effects seen on entry mediated by the glycoproteins from a virus (measles) that fuses and enters the cell through the plasma membrane. Interestingly, the trend was for greater reliance on functional GBA for viruses that enter through later, more-acidic endosomes (4, 43–46). We note that loss of GBA led to inhibition in single-cycle but not multicycle influenza virus infections (Fig. 4), suggesting that the perturbations to the sphingolipid pathway in our GBA KO cells may have consequences for IAV assembly or exit, findings that require further investigation. Indeed, a 2012 study demonstrated that the membrane of IAV may be enriched in sphingolipids, indicating a role for these lipids in the exit of the virus from host cells (47). On the basis of these findings, we hypothesized that GBA and optimal levels of GlcCer are required, in general, for proper trafficking of cargo along the endocytic pathway, particularly with respect to late endosomes. Indeed, we found that trafficking of not only influenza virus particles but also EGF and its receptor (to Lamp1^+^ endosomes) was impaired in GBA KO cells, which in the latter case correlated with significantly delayed degradation of EGFR. Collectively, our findings strongly suggest that GBA regulates normal trafficking of cargo along the endocytic pathway.

Often, altering the function of one enzyme in the sphingolipid pathway results in compensation that makes it challenging to pinpoint a single lipid species as the cause of a particular cellular phenotype (48). We found, by mass spectrometry analysis of lipids, that removal of GBA in both HEK 293 and A549 cells resulted in a significant increase in GlcCer mass. As such, we hypothesize that it was excess GlcCer and not changes in other sphingolipids that was responsible for the observed phenotypes. We did not note a decrease in ceramide levels, nor did we note major changes to nonglycosylated ceramide metabolites, such as sphingomyelin and sphingosine-1-phosphate (Fig. 3). We hypothesize this was due to a compensatory increase in *de novo* synthesis of ceramide from serine and palmitoyl-coenzyme A (CoA) (Fig. 1 and 3C). Indeed, increases in *de novo* synthesis may have been responsible for the increase in dihydrosphingosine that we observed in both GBA KO cell lines. We also noted an elevation in glucosylsphingosine levels, albeit the change in mass was much smaller than the change seen with GlcCer. Little is known about the metabolism of glucosylsphingosine, but several recent studies indicated that it may serve as a biomarker for Gaucher disease, a lysosomal storage disorder characterized by mutations in GBA (49–51). Our data suggest that GBA may catabolize both GlcCer and glucosylsphingosine (Fig. 3C), suggesting a more global role for GBA in sphingolipid biology and pathophysiology.

Even though we suggest that GBA is a major regulator of influenza infectivity, we cannot exclude other GlcCer metabolizing enzymes from consideration. A previous study showed that glucosylceramide synthase (UGCG) is required for infection by one type of bunyavirus (thrombocytopenia syndrome virus, causing severe fever) but not for another (Rift Valley virus) or for other enveloped viruses tested (VSV and EBOV) (19). Consistent with our work, those prior findings point to an optimal level of GlcCer being important for (certain) viral infections. And yet our findings extend the need for optimal levels of GlcCer to other, potentially many, enveloped viruses that enter cells through late endosomes as well as to important endogenous endocytic cargo, including EGF and its receptor.

It is well established that lipids are heterogeneously distributed throughout cells (52). Specialized lipid microdomains in various membrane compartments facilitate cellular organelle function and organization, including in endosomes (53). Sphingolipids in particular have been shown to be enriched in endosomes (54), and perturbations to the sphingolipid pathway result in abnormal endosome size, location, and function (55–58). GlcCer has been implicated in altering the physical properties of membranes; for example, increased GlcCer results in decreased membrane fluidity (59, 60). In addition, a recent paper demonstrated that cells taken from patients with Gaucher disease displayed restricted lateral lipid mobility and exhibited reduced rates of transferrin receptor endocytosis (61). Coupled with our findings, these reports suggest that the disruption in endocytosis that we observe in GBA KO cells for critical cargos, including pathogenic viruses and growth factor receptors, may be due in part to an alteration in the biophysical properties of cellular membranes. Hence, targeting GBA might prove beneficial as part of strategies to ameliorate viral infections that utilize the endocytic pathway.

## MATERIALS AND METHODS

### Cells.

HEK 293 (human embryonic kidney 293; ATCC CRL-1573), HEK 293T/17 (ATCC CRL-11268), A549 (human lung carcinoma; ATCC CCL-185), and BHK-21 (baby hamster kidney; ATCC CCL-10) cells were grown in Dulbecco modified Eagle medium (DMEM) supplemented with 10% fetal bovine serum (FBS), 1% sodium pyruvate, 1% antibiotic/antimycotic, and 1% l-glutamine at 37°C (all from Gibco Life Technologies) with 5% CO_2_.

### CRISPR/Cas9 gene editing.

Genomic RNA (gRNA) targeting GBA was selected using the CRISPR design tool developed by the Zhang laboratory at MIT and available at https://zlab.bio/guide-design-resources. The gRNA was cloned into a Cas9-sgRNA (Addgene plasmid catalog no. 68463; deposited by Su-Chun Zhang) using BbsI. The resulting plasmid, along with a plasmid encoding GFP, was cotransfected into HEK 293 and A549 cells and sorted for positive GFP expression into single cell colonies using an Influx flow cytometer. The cells, originally in wells of 96-well plates, were expanded, and the DNA from over 100 discrete colonies was extracted and analyzed by PCR and subsequent gel electrophoresis. PCR was performed using primers flanking the regions of interest and designed to produce fragments of ∼250 to 300 bp. Colonies with PCR products indicative of CRISPR activity (∼5 to ∼10 per cell line) compared to products from WT cells were maintained and analyzed by Western blotting (for GBA protein) and mass spectrometry (for sphingolipid content).

### Inhibitors and other reagents.

Epidermal growth factor (EGF) (catalog no. E9644) and bafilomycin A1 (catalog no. B1793) were purchased from Sigma-Aldrich. EGF-Alexa Fluor 555 (catalog no. E35350) was purchased from Thermo Fisher Scientific. BbsI was purchased from New England Biolabs (catalog no. R0539S).

### Influenza viruses and VLPs, VSV pseudoviruses, and EBOV trVLPs.

Stocks of PR8 IAV were obtained from Charles River Laboratories. PR8 NS-GFP was kindly provided by Thomas Braciale at the University of Virginia (62). All influenza viruses were grown in embryonated chicken eggs, thereby cleaving HA_0_, before any infection assays were performed (63, 64).

VSV-GFP pseudoviruses were produced using 5 × 10^5^ BHK-21 cells plated in 40 10-cm^2^ dishes and transfected at ∼75% to ∼80% confluence with plasmids encoding EBOV-GPΔ, measles virus F and HN, or VSV-G using polyethylenimine (PEI; Polysciences, Inc., catalog no. 23966). Measles F plasmid was generously provided by Yusuke Yanagi of Kyushu University (65). The next day, cells were infected with VSV-ΔG helper virus (from plaque eluate, with previously measured titer) encoding GFP for 1 h at 37°C, washed extensively with phosphate-buffered saline (PBS), and then cultured in growth medium overnight at 37°C. At 24 h postinfection with helper virus, cell supernatants containing budded pseudovirus were collected, centrifuged twice (1,360 × *g* for 10 min) to clear debris, and concentrated ∼50-fold using a Viva-Spin 20 300-kDa concentrator. Finally, the concentrated pseudovirus was centrifuged through a 20% sucrose cushion (in HEPES-MES [HM] buffer containing 20 mM HEPES, 20 mM MES [morpholineethanesulfonic acid], 130 mM NaCl, pH 7.4) in an SW28 rotor for 2 h at 112,398 × *g* at 4°C and then resuspended in 10% sucrose–HM. Pseudovirus stocks were stored at −80°C.

VSV-ΔG helper virus was produced as described previously (66). In brief, 5 × 10^5^ BHK-21 cells plated in five 10-cm^2^ dishes were transfected at ∼75% to ∼80% confluence with 12 μg (per dish) of plasmid expressing VSV-G using PEI. At ∼24 h later, the cells were infected with ∼40 μl of VSV-GFP plaque eluate (3.39 × 10^8^ infectious units/ml) in serum-free media for 1 h at 37°C. Cells were then washed extensively with PBS and incubated overnight in complete media at 37°C. The next day, supernatants containing helper virus were collected, centrifuged for 10 min at 1,070 × *g* to clear debris, and stored at −80°C.

Influenza virus M1-VLPs were produced by transfecting 1 × 10^6^ HEK 293T/17 cells in each of 5 10-cm^2^ dishes in complete media with no antibiotic/antimycotic using plasmids encoding βlaM1 and WSN HA plus WSN NA or VSV-G or EBOV-GPΔ using PEI. WSN is an H1N1 strain of influenza virus that is trypsin independent *in vitro* (67). The βlaM1 plasmid was kindly provided by Adolfo Garcia-Sastre and the NIAID Centers of Excellence for Influenza Research and Surveillance (CEIRS) program (68). Media containing VLPs were harvested 24 and 48 h posttransfection, pooled, and centrifuged twice to clear debris. The VLPs were then pelleted through a 20% sucrose cushion in HM buffer using an SW28 rotor for 2 h at 112,398 × *g* at 4°C and were then resuspended in 10% sucrose-HM. VLPs were stored at −80°C.

Transcription/replication-competent viral-like particles (trVLPs) were prepared as described previously (69). Briefly, HEK 293T/17 cells were transfected with pCAGGS-L (a tetracistronic minigenome plasmid), pCAGGS-VP35, pCAGGS-NP, pCAGGS-VP30, and pCAGGS-T7 polymerase. At 24 h posttransfection, the medium was replaced with fresh growth medium containing 5% FBS and cells were incubated at 37°C. At 72 h posttransfection, the medium was harvested, pooled, and centrifuged for 5 min at 800 × *g* to clear cellular debris.

### IAV reporter infection assay.

Cells were seeded in 96-well plates at a density of 3 × 10^4^ cells per well. The next day, cells were prechilled to 4°C for 15 min and then incubated with PR8 influenza virus encoding GFP fused to the N terminus of NS1 (multiplicity of infection [MOI] of ∼1) in growth medium without FBS or trypsin and centrifuged at 250 × *g* for 1 h at 4°C. The cells were then incubated at 37°C. At approximately 16 to 18 h postinfection, cells were lifted with trypsin, fixed in 4% paraformaldehyde (PFAM), and assayed for GFP signal on an Attune NxT flow cytometer. All GFP values were normalized to uninfected cells to account for any background signal.

### qPCR.

Cells were seeded in 24-well plates at a density of 5 × 10^4^ cells per well. The next day, cells were prechilled to 4°C for 15 min and then incubated with WT PR8 influenza virus in growth medium without FBS (with or without 1 μg/ml trypsin as indicated) and centrifuged at 250 × *g* for 1 h at 4°C. The cells were then incubated at 37°C. At the indicated time points, samples were harvested and RNA was extracted using TRIzol reagent according to the instructions of the manufacturer (Thermo Fisher Scientific catalog no.15596026). cDNA was generated using iScript cDNA synthesis (Bio-Rad catalog no. 1708891) according to the instructions of the manufacturer, and quantitative PCR (qPCR) was performed with the following primers: IAV M1 forward (5′-CTTCTAACCGAGGTCGAAACG-3′) and IAV M1 reverse (5′-GGCATTTTGGACAAAGCGTCTA-3′). Relative expression levels of IAV M1 mRNA were calculated after normalization to endogenous reference gene beta-2-microglobin (Bio-Rad catalog no. pHSACID0015347).

### Influenza virus M1-VLP entry assay.

Cells were seeded in 96-well plates at a density of 3 × 10^4^ cells per well. The next day, cells were prechilled to 4°C for 15 min and then incubated with influenza virus M1-VLPs (with previously measured titers) diluted in Opti-MEM I (OMEM) and centrifuged at 250 × *g* for 1 h at 4°C. The cells were incubated at 37°C for 3 h before addition of the βlaM substrate (CCF2-AM; Invitrogen catalog no. K1032) in loading buffer (phenol red-free DMEM, 5 mM [HEK 293] or 20 mM [A549] probenecid [MP Biomedicals catalog no. 156370], 2 mM l-glutamine, 25 mM HEPES, 200 nM bafilomycin) and were incubated for an additional hour at room temperature. Cells were then washed with PBS and allowed to incubate in loading buffer with 10% FBS overnight in the dark at room temperature. The following day, cells were lifted with trypsin, fixed in 4% PFAM, and analyzed for VLP entry as measured by CCF2-AM cleavage (resulting in fluorescence resonance energy transfer [FRET] disruption and a color shift from green [518 nm] to blue [447 nm]) on an Attune NxT flow cytometer.

### VSV pseudovirus infection assay.

Cells were seeded in 96-well plates at a density of 3 × 10^4^ cells per well. The next day, cells were prechilled to 4°C for 15 min and then incubated with VSV pseudoviruses in Opti-MEM I (OMEM) and centrifuged at 250 × *g* for 1 h at 4°C. Cells were then washed and incubated for 18 to 24 h at 37°C. The cells were then lifted, fixed, and analyzed for GFP expression via flow cytometry on an Attune NxT flow cytometer.

### trVLP infection assay.

Infection of HEK 293 cells by trVLPs was performed as described previously (69). Cells were seeded in opaque 96-well plates, and when the cells were approximately 50% confluent, they were transfected with (per well) 13.88 ng pCAGGS-Tim1, 4.16 ng pCAGGS-VP30, 6.94 ng pCAGGS-VP35, 6.94 ng pCAGGS-NP, and 55.55 ng pCAGGS-L in order to support entry and replication of infecting trVLPs. At 24 h posttransfection, the medium was removed and trVLPs were added. Cells were incubated for 18 to 24 h in growth medium at 37°C and then analyzed using a *Renilla*-Glo luciferase assay system (Promega catalog no. E2710) on a GloMax plate reader.

### Western blotting.

Cell samples were lysed with PBS containing 1% SDS, 5 mM EDTA, and 1 mM sodium vanadate (Sigma catalog no. S6508). These lysates were then resolved by SDS-PAGE and the proteins subsequently transferred to polyvinylidene difluoride (PVDF) membranes. The membranes were then probed with the indicated primary antibodies followed by secondary antibodies coupled to horseradish peroxidase. Signals were visualized following incubation with a chemiluminescent horseradish peroxidase (HRP) substrate. Images were captured with an Alpha-Innotech Fluorchem detector. For quantification, samples were normalized to signals for GAPDH (glyceraldehyde-3-phosphate dehydrogenase) in the lysates using Image Studio Lite.

### Antibodies.

The following antibodies were purchased from the indicated sources: anti-EGFR (A-10), Santa Cruz Biotechnology (catalog no. sc-373746); anti-GBA, Abcam (catalog no. ab55080); anti-GAPDH (14C10), Cell Signaling Technology (catalog no. 3683); anti-cathepsin B, Santa Cruz Biotechnology (catalog no. sc-365558).

### Lipid mass spectrometry.

Lipids were extracted from cell lysates and analyzed on an Acquity I-Class/Xevo TQ-S micro-IVD (*in vitro* diagnostic medical device) system as described previously (70). Mass spectrometry peaks were compared to internal standards, and all data are represented as picomoles of lipid per milligram of protein.

### EGFR degradation.

Cells were seeded in 6-well plates at a density of 6 × 10^5^ cells per well. The next day, cells were washed twice with PBS and then incubated with 50 ng/ml EGF in growth media at 37°C for the indicated times without the presence of cycloheximide. Cells were lysed and analyzed by Western blotting as described above. For quantification, samples were normalized to the signal for GAPDH and then to 0 h.

### Cathepsin activity assays.

*In vitro* cathepsin B and cathepsin L activities in cell lysates were measured as described previously (71, 72). Briefly, cathepsin L activity was assayed with cathepsin B+L substrate Z-Phe-Arg-7-AMC (Calbiochem catalog no. 03-32-1501) in the presence of 1 μM CA-074 (Calbiochem catalog no. 205530), a cathepsin B inhibitor. Cathepsin B activity was measured in the same manner using Z-Arg-Arg-7-AMC (Calbiochem catalog no. 219392) and no inhibitor. *In vivo* cathepsin B activity was measured using Magic Red-(RR)_2_ (Bio-Rad catalog no. ICT937) stain per the manufacturer’s instructions.

### Influenza virus fusion assay.

Influenza PR8 virus was dually labeled with 3,3′-dioctadecyloxacarbocyanine (DiOC18) and octadecyl rhodamine B (R18) as described previously (31, 48) at final concentrations of 0.2 and 0.4 μM, respectively. The reaction mixture was subjected to vigorous vortex mixing and left to incubate for 1 h at room temperature before being filtered through a 0.22-μm-pore-size filter. Labeled virus particles were then bound to prechilled cells at an MOI of ∼5 (by pretitration visual inspection) at 4°C for 15 min. Following binding, cells were washed three times with cold PBS before being placed at 37°C for 40 min. Cells were then fixed in 4% paraformaldehyde for 20 min and imaged. Images were acquired on a Nikon Eclipse TE2000-E microscope equipped with a Yokogawa CSU 10 spinning-disk confocal unit and a 512-by-512-pixel Hamamatsu 9100c-13 EM-BT camera using a 60×/1.45 numerical aperture (NA) Nikon Plan APO Apo TIRF oil immersion objective. Nonfused influenza virus particles appeared red, as the green signal of DiOC18 (emission [Em], wavelength of 501 nm) was suppressed by a combination of self-quenching and FRET from DiOC18 to R18 (Em, 578 nm). In contrast, fused particles appeared green due to loss of FRET and self-quenching. Images were processed for Gaussian background subtraction and then by automated particle counting for the numbers of red and green particles using ImageJ. The number of green particles was then divided by the number of red particles to obtain the reported ratio of fused to nonfused events for each field.

### Trafficking assays.

Cells were transfected with GFP-Lamp1 (Addgene plasmid catalog no. 34831, deposited by Esteban Dell’Angelica) using Lipofectamine 2000 (Invitrogen catalog no. 11668-030) and incubated overnight in growth media. The next day, PR8 influenza virus was incubated with 1 μM R18 for 1 h at room temperature as described previously (73). Labeled viruses were filtered through a 0.22-μm-pore-size filter and then immediately bound to prechilled cells at an MOI of >1 at 4°C for 15 min. For analysis of EGF trafficking, Alexa Fluor 555-EGF was bound to prechilled cells at 4°C for 15 min at a final concentration of 100 ng/ml. Following binding with either fluorescently labeled IAV or fluorescently labeled EGF, cells were washed three times with PBS followed by the addition of prewarmed media lacking IAV or EGF and placed at 37°C for 40 min. Cells were then fixed in 4% paraformaldehyde containing 5 μg/ml of Hoechst 33342 (Thermo Fisher Scientific catalog no. H3570) for 20 min before imaging was performed. Images were acquired on a Nikon Eclipse TE2000-E microscope equipped with a Yokogawa CSU 10 spinning-disk confocal unit and a 512-by-512-pixel Hamamatsu 9100c-13 EM-BT camera. Samples were acquired using a 100×/1.45 numerical aperture (NA) Nikon Plan Apo TIRF oil immersion objective.

To quantify colocalization of IAV or EGF with Lamp1, 100 independent images per experiment from two independent experiments were captured. Each image was uniformly processed for Gaussian background subtraction and then for the Mander’s coefficient of colocalization of IAV or EGF with Lamp1 using the automated JACoP plugin in ImageJ. To quantify the number of IAV or EGF particles, each image was uniformly processed for Gaussian background subtraction and then particles were counted using the automated particle analysis tool in ImageJ.

### Measurement of (endo)lysosomal pH.

The pH of lysosomes was measured using an FITC-dextran conjugate as described previously (43). Cells were plated at a density of 9,000 cells/cm^2^ in 35-mm-diameter dishes and incubated in cell culture medium containing 0.1 mg/ml FITC-dextran for 72 h. Cells were then pulsed in medium without FITC-dextran for 2 h, lifted by trypsinization, and washed with PBS. Cells were then resuspended in PBS and analyzed on a BD four-color FACSCalibur flow cytometer by excitation with a 488-nm-wavelength laser, and emission data were collecting at 530 nm (FL1) and 610 nm (FL2). The FL1/FL2 ratios of samples were compared to a standard curve generated using cells incubated with pH-calibrated Britton-Robinson buffers containing 50 mM sodium azide, 50 mM 2-deoxyglucose, and 10 μM nigericin.
